# Tobacco Asthma

**Published:** 1887-03

**Authors:** 


Tobacco Asthma.—Russo Gilberti reported to the *Società d' Igiene* numerous cases of functional disorders caused by tobacco, among which is the following: A young man 24 years of age, well developed and nourished, but of an erethistic temperament and a hereditary tendency to convulsions, was seized with severe attacks of asthma which he attributed to smoking. His physician advised him to discontinue the use of tobacco and avoid rooms where there was tobacco smoke, and for more than a year he has not had the slightest attack of asthma.

This case confirms the opinion of Peter, who considers tobacco a true poison to the pneumogastric, and may even in small doses injure those who are especially susceptible to its influence.—*Lo Sperimentale.*

